# Beneficial Effect of Statin Therapy on Arterial Stiffness

**DOI:** 10.1155/2021/5548310

**Published:** 2021-03-30

**Authors:** Mona Alidadi, Fabrizio Montecucco, Tannaz Jamialahmadi, Khalid Al-Rasadi, Thomas P. Johnston, Amirhossein Sahebkar

**Affiliations:** ^1^Department of Nutrition, Faculty of Medicine, Mashhad University of Medical Sciences, Mashhad, Iran; ^2^IRCCS Ospedale Policlinico San Martino Genoa-Italian Cardiovascular Network, 10 Largo Benzi, 16132 Genoa, Italy; ^3^First Clinic of Internal Medicine, Department of Internal Medicine, University of Genoa, 6 viale Benedetto XV, 16132 Genoa, Italy; ^4^Department of Food Science and Technology, Quchan Branch, Islamic Azad University, Quchan, Iran; ^5^Medical Research Centre, Sultan Qaboos University, Muscat, Oman; ^6^Division of Pharmacology and Pharmaceutical Sciences, School of Pharmacy, University of Missouri-Kansas City, Kansas City, MO 64108, USA; ^7^Biotechnology Research Center, Pharmaceutical Technology Institute, Mashhad University of Medical Sciences, Mashhad, Iran; ^8^Applied Biomedical Research Center, Mashhad University of Medical Sciences, Mashhad, Iran; ^9^School of Pharmacy, Mashhad University of Medical Sciences, Mashhad, Iran

## Abstract

Arterial stiffness describes the increased rigidity of the arterial wall that occurs as a consequence of biological aging and several diseases. Numerous studies have demonstrated that parameters to assess arterial stiffness, especially pulse-wave velocity, are predictive of those individuals that will suffer cardiovascular morbidity and mortality. Statin therapy may be a pharmacological strategy to improve arterial elasticity. It has been shown that the positive benefits of statin therapy on cardiovascular disease is attributable not only to their lipid-lowering capacity but also to various pleiotropic effects, such as their anti-inflammatory, antiproliferative, antioxidant, and antithrombotic properties. Additionally, statins reduce endothelial dysfunction, improve vascular and myocardial remodeling, and stabilize atherosclerotic plaque. The aim of the present review was to summarize the evidence from human studies showing the effects of statins on arterial stiffness.

## 1. Introduction

Arterial stiffness, as an indicator of structural and functional changes in the arterial wall, occurs as a result of the aging process and the consequence of several diseases such as metabolic syndrome, hypertension, hypercholesterolemia, and chronic inflammatory disease [1–3]. Arterial stiffness is defined as the decreased ability of an arterial wall to dilate and contract in response to an alteration in intraluminal pressure [4]. This phenomenon is thought to have multifactorial pathophysiology, including disturbances in hormone levels, inflammatory responses, oxidative balance, decreased nitric oxide (NO) bioavailability, and increased shear stress induced by risk factors such as hyperlipidemia and hypertension [5–7].

Arterial stiffening is accompanied by several important processes, including the breakage and reduction of elastin fibers, increases in collagen deposition, transforming growth factor- (TGF-) *β*, intercellular adhesion molecules (ICAM), and proinflammatory cytokines. Additionally, arterial stiffness is also accompanied by the accumulation of advanced glycation end-products (AGEs), activation of matrix metalloproteinases (particularly MMP-2), and infiltration of smooth muscle cells, macrophages, and mononuclear cells [1, 3, 8, 9]. This remodeling and reduction of elastic properties (stiffening) at the level of elastic arteries is called arteriosclerosis [10]. Though there are many parameters for evaluation of vascular stiffness, the most common are pulse-wave velocity (PWV), augmentation index (AIx), augmentation pressure (AP), cardio-ankle vascular index (CAVI), arterial compliance, transit time of the reflected wave (Tr), stiffness index (SI), and reflection index (RI) [11–16]. Among the various parameters, carotid-femoral PWV (cf-PWV), a direct measure of aortic stiffness, is the most popular method to use, because of its relative ease in evaluation, relatively inexpensive cost, reliability, safety, noninvasiveness, and, most importantly, because of the strong body of evidence confirming its association with increased risk for an initial cardiovascular event [17–19]. It has been demonstrated that elevated PWV is a strong predictor of cardiovascular incidence and mortality, independent of traditional risk factors [20–25].

Treatment strategies to improve arterial elasticity involve pharmacological and nonpharmacological methods (such as weight loss, restriction in salt intake, smoking cessation, and increasing aerobic physical activity). Though pharmacological interventions are mainly based on antihypertensive drugs, numerous studies have revealed the protective effects of statins, also known as HMG-CoA reductase inhibitors, against vascular stiffening [1, 26]. Several investigations have demonstrated a strong association between lipid levels (high TG, high LDL-cholesterol, non-HDL-cholesterol, high TG/HDL-C, TC/HDL-C ratio, etc.) and arterial stiffness. Kim et al. reported a modest increase in arterial stiffness due to dyslipidemia only in women [27]. In another study, Mozos et al. revealed that increased LDL-cholesterol levels correlated with central systolic and diastolic blood pressure, PWV, and pulse pressure amplification [28]. Moreover, it was shown that non-HDL-cholesterol, TG, and the TC/HDL-cholesterol ratio were consistently associated with arterial stiffness independent of LDL-cholesterol in a Chinese population [29]. In addition to the LDL-lowering effect of statins, given their pleiotropic effects such as anti-inflammatory and antioxidant capabilities, this class of lipid-modifying agents may play a beneficial role in arterial stiffness [5, 7, 30–34].

This paper is aimed at summarizing evidence from human studies (Table 1) describing the effects of statins on arterial stiffness and providing an overview of proposed mechanisms that are thought to mediate this effect.

## 2. Simvastatin

The effect of simvastatin on arterial elasticity has been assessed in a number of clinical trials. In a randomized, double-blind, placebo-controlled study, the protective effect of simvastatin against arterial stiffness induced by inflammation (by typhoid vaccine) was evaluated in 50 healthy volunteers. In this study, aortic PWV, as well as flow-mediated dilatation (FMD), was assessed three times, at baseline, following 14 days of treatment with either simvastatin (40 mg/day) or placebo, and 8 h after a typhoid vaccination on the morning of day 15. The aortic PWV of the placebo group was significantly elevated following vaccination (5.80 ± 0.87 (baseline) vs. 6.21 ± 0.97 m/s (after vaccination on day 15), 95% CI 0.19, 0.62, *P* = 0.002). However, the aortic PWV was significantly reduced in the simvastatin group (5.68 ± 0.73 (baseline) vs. 5.72 ± 0.74 m/s (after vaccination on day 15), 95% CI 0.19, 0.27, *P* = 0.9). Interestingly, typhoid vaccine did not affect AP, AIx, mean arterial pressure, or brachial PWV in either group [35]. Also, in a 6-week randomized, parallel-group, placebo-controlled study, 64 subjects with confirmed chronic obstructive pulmonary disease (COPD) were enrolled and randomized to ingest either simvastatin (20 mg/day) or placebo. In this trial, patients with high baseline aortic PWV (PWV > 10 m/sec, *n* = 22) exhibited a statistically significant improvement in aortic PWV in comparison to placebo (-2.8 m/sec, *P* = 0.03) [36]. In another trial, 51 patients with primary arterial hypertension and high-normal serum cholesterol levels were randomized to receive simvastatin (20 mg/day) or placebo, each for 6 months. A significant improvement was observed in PWV values of the simvastatin-treated group (from 13.21 ± 1.83 m/s initially to 9.18 ± 0.59 m/s, *P* < 0.01), while this trend was nonsignificant in the placebo group (12.98 ± 1.61 m/s initially vs. 11.84 ± 1.19) [37].

In a different randomized, double-blind, cross-over trial, 100 participants with a history of statin myalgia were assigned to consume daily 20 mg simvastatin, or matched placebo, for 8 weeks. In this study, vascular stiffness indices included central PWV, peripheral PWV, AIx, AP, and central arterial pressure and were evaluated before and after each treatment. At the end of the study, the central PWV significantly improved, compared with baseline, following simvastatin treatment (*P* = 0.01) [38]. In yet another double-blind, cross-over study, Shige et al. assessed the effects of simvastatin (20 or 40 mg/day) therapy versus placebo on arterial compliance and central and peripheral PWV in 20 hypercholesterolemic subjects. After 4 weeks of simvastatin treatment, peripheral PWV significantly decreased compared to placebo (9.40 ± 1.30 vs. 10.12 ± 1.27, *P* = 0.028) [39]. An additional cross-over study, which included 20 patients with active rheumatoid arthritis, randomly assigned the patients to receive either 20 mg/day simvastatin or 10 mg ezetimibe, for 6 weeks. Among arterial stiffness parameters (aortic PWV, AIx, and AP), PWV substantially improved following both treatments, even though ezetimibe is technically not classified as a statin drug [40].

In a nonrandomized study, forty hypercholesterolemic men and women were divided into four groups. Group 1 consisted of previously untreated patients who received simvastatin at 40 mg/day; group 2 was comprised of patients previously treated with 40 mg/day simvastatin but received simvastatin at 80 mg/day during the study; group 3 was previously untreated patients who received ezetimibe at 10 mg/day; and group 4 included patients previously treated with 40 mg/day simvastatin, and who continued to receive simvastatin at 40 mg/day, together with 10 mg/day of ezetimibe. After 3 months of treatment, only the patients in group 1 (simvastatin 40 mg/day) showed a significant (*P* < 0.001) reduction in AIx (30.2 ± 8.3% (baseline; i.e., prior to treatment) to 21.6 ± 6.5% (after 3 months of treatment)] [41]. In a study by Gepner et al. it was reported that simvastatin may have a temporary influence on aortic stiffness. In this trial, 88 middle-aged adults at increased risk for dementia were randomly assigned to receive simvastatin (40 mg/day), or matched placebo, for 18 months. By 12 months, the AIx was significantly reduced when compared to the placebo group (-2.3% change from baseline (statin group) vs. 1.2% change from baseline (placebo group), *P* = 0.007), but at the end of 18 months, no significant difference was observed between the groups (-1.1% change from baseline (statin group) vs. 0.2% change from baseline (placebo group), *P* = 0.3) [42]. Thus, Gepner et al. suggested that the decrease in aortic stiffness due to simvastatin treatment was only transient. Along these lines showing little benefit to simvastatin treatment as it pertains to aortic stiffness, Balaguer et al. conducted a double-blind, randomized, placebo-controlled trial with 18 chronic obstructive pulmonary disease (COPD) patients. The patients were divided to receive either placebo or simvastatin (40 mg/day), for 12 weeks. At the end of the trial, no significant changes were observed in parameters used to assess arterial stiffness, including PWV and AIx [43]. As further validation of the results obtained by Balaguer et al. mentioned directly above, Cash et al. conducted a single-blind, randomized controlled trial with 13 primary biliary cirrhosis patients with hypercholesterolaemia. The 13 patients were randomly assigned to two groups: the first group received 20 mg/day simvastatin, while the second group received placebo at the same dose. After 12 months, these authors found no significant changes in parameters used to assess arterial stiffness (aortic PWV, AIx75, and Tr) between the two groups [44].

## 3. Atorvastatin

To evaluate the efficacy of atorvastatin on improving arterial stiffness, several studies have been performed, which can be divided into single-arm and two-arm trials.

### 3.1. Single-Arm Trials

In a study by Wang et al. it was reported that six months of atorvastatin treatment (20 mg/day) resulted in a significant reduction in baPWV (10.63%, *P* < 0.01) in hypertensive patients [45]. Additionally, it was previously reported that atorvastatin administration (10 mg/day) for 6 months decreased the femoral-ankle PWV of 22 patients with type-2 diabetes and hypercholesterolemia (from 1123 ± 162 cm/s to 1073 ± 142 cm/s, *P* = 0.019). However, in this study, other indices of vascular elasticity (i.e., heart-carotid, heart-brachial, and heart-femoral PWV) did not change with atorvastatin treatment [46]. In another study, 20 mg/day atorvastatin was given to 37 systemic lupus erythematosus female patients for 8 weeks. At the end of the treatment, aortic PWV was unaffected by the treatment, but a significant improvement was observed in patients with baseline pathological arterial stiffness, which was maintained for six months following cessation of atorvastatin treatment [47].

In a trial of rheumatoid arthritis patients, AIx markedly decreased after 6 and 12 weeks of daily intake of 20 mg atorvastatin. Those participants with the highest scores for disease severity demonstrated the maximum improvement in arterial stiffness [48]. Ratchford et al. reported that 30 days of atorvastatin therapy (80 mg/day) in stroke-free and statin-naive subjects led to a significant improvement in the stiffness index (SI) [49]. Similarly, receiving 20 mg/day atorvastatin for 2 years in patients with hypercholesterolemia led to a significant amelioration in aortic SI by 14% (*P* = 0.019) [50].

In two trials conducted by Leibovitz et al., the effect of atorvastatin on arterial compliance was evaluated. The first study showed that after 20 weeks of statin treatment (mean dose: 16 ± 1.6 mg/day), small artery compliance substantially increased in 17 patients with severe hypercholesterolemia [51]. The results of the second study indicated that after adding atorvastatin (10 or 20 mg/day, according to the investigator's decision) to amlodipine for a 3-month period, small artery compliance noticeably increased in hypertensive, hyperlipidemic patients [52]. In addition, following 6 months of treatment with 40 mg/day atorvastatin, small and large artery compliance in 33 patients with coronary artery disease (CAD) was significantly improved [53]. To evaluate the protective effect of a high, single dose of atorvastatin against arterial stiffening, Tulmaç et al. conducted a trial with 30 dyslipidemic patients without atherosclerosis. In this trial, 24 hours after ingestion of atorvastatin (80 mg), arterial stiffness parameters (SI and RI) were found not to be altered [54]. Finally, following 12 months of atorvastatin treatment (10 mg/day) in 30 hypercholesterolemic subjects, no substantial changes were observed in the baPWV [55].

### 3.2. Two-Arm Trials

In a prospective, nonrandomized study, 79 subjects with type-2 diabetes and dyslipidemia were recruited to evaluate whether 12 months of the Mediterranean diet alone (diet group), or 10 mg atorvastatin plus the Mediterranean diet, would affect arterial stiffness parameters. The atorvastatin-treated group showed a significant improvement in aortic PWV compared to baseline and the subjects in the “diet group.” Additionally, carotid-radial PWV showed a reduction, but not a statistically significant trend during assessments at 3, 6, and 12 months [56]. In another study, 63 CAD patients with hyperlipidemia received either 10 mg of atorvastatin daily plus a low-fat diet or a low-fat diet alone (diet group). After 6 months, the PWV values (carotid-femoral, carotid-radial, and carotid-distal) for the subjects in the atorvastatin plus low-fat diet group were markedly decreased compared to baseline values and corresponding values for the patients contained in the “diet group” [57].

In a randomized study, Huang et al. reported that 20 mg/day atorvastatin in combination with levamlodipine besylate in hypertensive patients with dyslipidemia and quantifiable biomarkers indicating an overall inflammatory environment exhibited a markedly improved PWV compared to the group treated with levamlodipine besylate only following 8 weeks of intervention (*P* < 0.05) [58]. In another different randomized, double-blind, placebo-controlled study, indices of vascular stiffness, including aortic PWV, SI, and arterial compliance (but not brachial PWV), were assessed following 12 weeks of daily intake of atorvastatin (80 mg) in overweight and obese middle-aged and older adults. This particular study showed a significant improvement in the indices of vascular stiffness for subjects taking atorvastatin when compared to those subjects in the placebo group [59]. In a similar design, Kanaki et al. showed that administration of atorvastatin at the dose of 10 mg/day for 6 months in hypertensive and hypercholesterolemic patients led to a substantial decrease in the values of aortic PWV, normalized PWV (for mean blood pressure (MBP) and heart rate), AIx75, and AP compared to these same parameters for patients in the placebo group [60]. In another parallel, double-blind clinical trial, 142 hypertensive patients were randomized to receive atorvastatin (10 mg/day), or placebo, for 12-18 months. At the end of the study, atorvastatin treatment was associated with a significant improvement in arterial stiffness parameters (carotid AIx, carotid AP, and RI from the body) compared to these same parameters for patients in the placebo group [61].

In a randomized, open-label trial lasting 12 months, 51 male patients with type-2 diabetes and microalbuminuria were divided into two groups according to the treatment dose of atorvastatin (10 or 80 mg/day). At 3- and 12-month follow-ups, carotid-femoral PWV improved in both groups (*P* < 0.001 from baseline), although there was no difference between the 10 mg or 80 mg dose (i.e., there was no dose-dependent effect) between the groups [62]. In a similar study, 51 subjects with moderate cholesterolemia (total cholesterol: 200-250 mg/dl) were randomly assigned to orally ingest different doses of atorvastatin (10 or 40 mg/day). Carotid-radial PWV was measured at the start of the study (baseline), and again at one and eight weeks. The results of this study showed that the high-dose treatment (40 mg/day) significantly decreased PWV after one and eight weeks, and the change in the PWV at eight weeks was significantly different between the two groups [63].

In one double-blind, cross-over trial, administration of atorvastatin 40 mg/day for 4 weeks in patients with ischemic heart failure was associated with a substantial reduction of AIx75 compared with baseline, as well as Aix75 values for patients who received 10 mg/day atorvastatin (−2.88 ± 6.38% vs. −0.86 ± 4.09%, *P* = 0.048) [64]. Furthermore, in a different double-blind trial, 20 hypercholesterolemic patients were randomly assigned to receive either 20 or 80 mg/day atorvastatin for 3 months. At the end of the intervention, the distensibility coefficient increased significantly in both groups (*P* < 0.001), but there was not a significant difference in the distensibility coefficient between the groups [65].

In a randomized trial involving atorvastatin treatment (10 mg/day) in 57 normotensive, normolipidemic individuals with type-2 diabetes, it was reported that atorvastatin therapy significantly lowered the values of baPWV compared to baseline (1712.03 ± 349.9 cm/s vs. 1558.81 ± 303.26 cm/s); however, this alteration was not statistically significant in the placebo group (1692.03 ± 425.15 cm/s vs. 1636.78 ± 425.1 cm/s) [66]. Kabaklić and Fras, in a double-blind, placebo-controlled clinical trial, evaluated atorvastatin treatment and arterial stiffness in patients with angina pectoris and a normal coronary angiogram. Patients were randomly divided to receive 20 mg atorvastatin, or matched placebo. After a 6-month intervention, although AIx75 significantly improved in the atorvastatin group, there was no noticeable difference observed between the groups (-114.49% statin group vs. -30.77% placebo group, *P* = 0.077) [67].

Fassett et al. reported that there was a rising trend in aortic PWV in patients with chronic kidney disease (CKD) even after atorvastatin administration (10 mg/day) for 3 years. In this investigation, although the rising trend of aortic PWV was 41% slower in the atorvastatin-treated group, no significant difference was detected between the groups. Moreover, other arterial parameters (AIx and arterial pressure) were unaffected by the intervention [68]. Joyeux et al. also conducted a randomized, double-blind, parallel clinical trial and reported that a daily intake of 40 mg atorvastatin, or matched placebo, for 12 weeks did not affect aortic PWV of subjects suffering from obstructive sleep apnea syndrome [69]. Additionally, in a cross-over, placebo-controlled study, the effect of daily intake of atorvastatin (40 mg/day) was assessed in first-degree relatives of patients with premature coronary artery disease and endothelial dysfunction. After a 6-week intervention, no significant effect on AIx was noted [70].

Lastly, in another double-blind, placebo-controlled trial, 23 patients with hypertension and hypercholesterolemia were recruited to receive either 10 mg/day atorvastatin or placebo, for 12 weeks. At the end of the 12-week intervention, indices of arterial stiffness (aortic PWV, carotid AIx) were not modified as a result of the intervention, even after adjustment for age, gender, and basal mean blood pressures (*P* ≥ 0.05) [71]. In a similar double-blind study, 325 subjects with concomitant hypertension and dyslipidemia were assigned to orally ingest either 10 mg/day atorvastatin or placebo. After 8 weeks of treatment, no statistically significant difference in compliance of small and large arteries was detected [72].

## 4. Rosuvastatin

Rosuvastatin, as another member of the statin family of drugs that inhibit HMG-CoA reductase, has been evaluated in several arterial stiffness trials.

### 4.1. Single-Arm Trials

In one particular study, rosuvastatin was administered to 114 patients with arterial hypertension and classified as having high and very high cardiovascular risk. These were patients who did not reach their target level of cholesterol after 1 year of atorvastatin treatment (20 mg/day). Rosuvastatin was initiated at a dose of 10 mg/day and then titrated upward to 20 and 40 mg/day if the cholesterol goal was not obtained. After 1.5 years of treatment, CAVI and AIx were improved by 12% and 17%, respectively [73]. Another investigation assessed the effect of 5-10 mg/day rosuvastatin treatment on arterial stiffness markers (aortic PWV, AIx, AIx75, and arterial pressure) in 20 subjects with newly diagnosed, heterozygous familial hypercholesterolemia. This investigation lasted for 3 months, and ultimately, aortic PWV showed a significant improvement (from 8.30 ± 1.4 to 7.13 ± 0.97 m/sec, *P* < 0.0001), even after adjusting for mean blood pressure and heart rate (*P* < 0.0001) [74].

In two studies, Ikdahl et al. assessed the efficacy of rosuvastatin treatment (20-40 mg/day) on parameters of arterial stiffness in patients with inflammatory joint diseases. In the first study, the values of aortic PWV and AIx did not change after 18 months of rosuvastatin treatment (*P* value: 0.78 and 0.06, respectively). In this study, flow-mediated dilation (FMD), an endothelial dysfunction index, was significantly improved, and the reduced AIx was significantly correlated with *Δ*FMD (*P* < 0.05) [75]. In the second study, after 18 months of rosuvastatin intervention, the aortic PWV, AIx, and brachial BP were significantly decreased. Additionally, the baseline AIx and aortic PWV values (greater arterial stiffness) were associated with a more marked reduction during the study (*P* < 0.001) [76].

In a final one-arm study, 28 patients with systemic sclerosis were enrolled to receive 20 mg rosuvastatin daily for 6 months. At the end of this trial, none of the vascular stiffness parameters (aortofemoral and carotid-femoral PWV) was improved [77]. Lastly, Deguchi et al. determined that there was no significant improvement in CAVI of dyslipidemic patients with cerebral infarction who received 5 mg/day rosuvastatin for 12 months [78].

### 4.2. Two-Arm Trials

In a randomized, open-label study, seventy-one patients with primary hypercholesterolemia were divided into two groups; the first group received 10 mg/day rosuvastatin with a low-fat diet, and the second group received only a low-fat diet. This intervention lasted for 4 weeks, and, at the conclusion of the study, the aortic PWV showed a significant improvement in the rosuvastatin group (from 9.5 ± 1.9 m/s to 7.8 ± 1.5 m/s, *P* = 0.02), while no substantial change was detected in the low-fat diet group (from 9.2 ± 2.1 m/s to 8.8 ± 1.9 m/s) [79]. In another randomized, open-label study, Igase et al. showed that administration of rosuvastatin (2.5 mg/day, which was accompanied by diet and exercise) in postmenopausal women with dyslipidemia for 3 and 12 months significantly decreased the levels of baPWV and SI compared to the control group (diet and exercise alone) [80]. In yet another double-blind, placebo-controlled, cross-over study, 29 hypercholesterolemic patients were randomized to orally ingest either rosuvastatin (10 mg/day) or placebo, for 42 days. After the intervention, the AIx75 significantly improved compared to the placebo group [81].

To evaluate the efficacy of low dose (5 mg/day) versus high dose (20-40 mg/day) of rosuvastatin on arterial elasticity, 40 patients with optimally controlled arterial hypertension were enrolled and followed for 6 months. At the end of the study, aortic PWV and AIx75 improved in both groups, and the high dose was more effective in reducing the PWV [82]. Additionally, a study was conducted wherein 120 hyperlipidemic subjects received either 10 mg/day rosuvastatin or no statin. After 12 weeks of drug intervention, the values of baPWV and radial AIx significantly improved in the rosuvastatin-treated group (baPWV: 1340 ± 177 vs. 1477 ± 159 cm/s; radial AIx: 44 ± 13% vs. 57 ± 15%; *P* < 0.01 and *P* < 0.05), while no substantial changes were observed in the nonstatin treatment group [83].

Lastly, in a randomized, double-blind, placebo-controlled clinical trial, 42 patients with rheumatoid arthritis received rosuvastatin treatment (10 mg/day) for 1 year. However, no significant changes were observed in the values of AIx [84].

## 5. Pitavastatin

With regard to the effects of pitavastatin on arterial stiffness, we identified two studies in the literature. In the first study, forty-eight hypercholesterolemic patients received pitavastatin 4 mg/day, and arterial stiffness parameters (stiffness index-*β*2 and SI) were assessed at baseline and at a 3-month follow-up. At the end of the study, the stiffness index-*β*2 significantly improved in patients taking 4 mg/day of pitavastatin [85]. In the second study, Miyashita et al. reported that after 12 months of pitavastatin treatment (2 mg/day), CAVI was markedly reduced (from 9.54 to 8.91) in 45 patients with type-2 diabetes and hyperlipidemia [86].

## 6. Pravastatin

Only a few studies have examined the efficacy of pravastatin on arterial stiffness parameters. In one such study, 59 hypercholesterolemic patients were recruited to orally ingest pravastatin (10 mg/day) for 6 months. At the end of the study, the PWV significantly improved in those patients who were documented to have a higher reduction in TC (≥15%). In addition, PWV reduction remained over a 5-year period [87].

In another study, pravastatin treatment (10 mg/day) for 6 months in 15 hyperlipidemic patients significantly improved baPWV, while the values increased, but were not statistically significant, in the healthy control volunteers [88]. Lastly, Duan et al. reported that pravastatin therapy (5-10 mg/day) for six months improved endothelial dysfunction in 13 children with medium to giant coronary aneurysms due to Kawasaki disease, although it did not affect the value of SI when compared to this same parameter in healthy control children [89].

## 7. Fluvastatin

Fluvastatin is another member of the statin drug class and has been used to treat arterial wall rigidity in a number of trials.

### 7.1. Single-Arm Trials

To evaluate the impact of fluvastatin on arterial elasticity, 60 hypercholesterolemic subjects with, or without coronary artery disease, received fluvastatin at a dose of either 40 or 80 mg/day, based on the percent of cholesterol reduction during the first 12 weeks. In this study, aortic compliance markedly improved following 1 year of statin treatment (from 11.72 ± 5.4 to 16.49 ± 7.3 *μ*l/mmHg, *P* < 0.0001) [90]. However, Ersoy et al. reported that 80 mg/day fluvastatin administration for 6 months did not alter large and small vessel compliance in 14 dyslipidemic renal transplant recipients [91].

### 7.2. Two-Arm Trials

As it pertains to two-arm trials evaluating the potential benefit of fluvastatin for arterial stiffness, one double-blind, placebo-controlled trial enrolled 22 diabetic ESRD patients with normal serum lipid levels. These patients were randomly assigned to orally ingest fluvastatin (20 mg/day), or placebo, for 6 months. During the study, the PWV exhibited a progressive reduction in the fluvastatin group and a progressive elevation in the placebo group, and ultimately (at 6 months), the difference between the groups was statistically significant [92]. In another randomized, double-blind, placebo-controlled trial, fluvastatin administration at a dose of 10 mg/day for 14 and 30 days in apparently healthy, middle-aged males substantially improved arterial stiffness parameters (PWV and SI) compared with the placebo group [93]. In this trial, five months after discontinuation of fluvastatin treatment, the SI was still markedly improved when compared to baseline values (*P* = 0.01). In addition, there was an investigation in which forty hyperlipidemic subjects were enrolled in an open-label study and were randomized to receive either 40 mg/day fluvastatin or diet (control group), for 12 months. In this investigation, the baPWV noticeably decreased in the fluvastatin group, while it increased in the control diet group. Moreover, fluvastatin treatment led to a significant improvement in the SI when compared to baseline values [94]. Finally, in a randomized, single-blind trial, 93 subjects with CAD and hyperlipidemia were assigned to receive either fluvastatin (20-40 mg/day) or bezafibrate (200-400 mg/day) for 5 years. In this trial, it was noted 4that the fluvastatin-treated group showed a decreasing trend in the values of the baPWV, while this trend was shown to increase in the bezafibrate-treated group. Importantly, the difference in the values of the baPWV between the groups was statistically significant after 12 months [95].

## 8. Comparison of Different Statins

In one particular study, 82 patients with arterial hypertension and dyslipidemia were divided to receive either atorvastatin or rosuvastatin. After five weeks of intervention, a significant reduction in SI values and a nonsignificant improvement in AIX and RI were observed in both groups [96].

There have been other trials in which the efficacy of one statin was compared with another statin as it pertains to arterial stiffness parameters. For example, in one controlled trial, 36 participants with coronary artery disease were randomized to orally ingest either rosuvastatin (10 mg daily) or simvastatin/ezetimibe (10/10 mg/day) for 8 weeks. In this trial, the value of the baPWV did not change in the simvastatin/ezetimibe group, while it improved significantly in the rosuvastatin-treated group when compared with baseline values (from 1772 ± 430 cm/s to 1603 ± 398 cm/s) and baPWV values for the simvastatin/ezetimibe group [97]. In another clinical trial conducted by Ilyukhin et al., 38 ischemic heart disease patients were administered either 10 mg/day atorvastatin or 20 mg/day simvastatin, for 6 months. At the end of the study, atorvastatin and simvastatin treatment resulted in a 10.05% and 4.66% improvement in PWV, respectively [98].

In addition, an open-label clinical study was conducted that involved 75 patients with high-risk cardiovascular disease and dyslipidemia. The patients were randomized to take either rosuvastatin (2.5-5 mg/day) or fluvastatin (20-40 mg/day), for 12 months. In this trial, statin treatment significantly improved the values of baPWV, but rosuvastatin was more effective than fluvastatin [99]. Moreover, in a randomized, single-blind trial, the effect of daily oral administration of 5 mg simvastatin, 20 mg fluvastatin, 10 mg pravastatin, and a nonstatin antihyperlipidemic drug was assessed in 85 hyperlipidemic, hypertensive subjects. At the end of the 12-month study, only fluvastatin treatment in hyperlipidemic, hypertensive subjects led to a significant reduction in the values of baPWV when compared to baseline values and corresponding values of baPWV for the other groups [100].

## 9. Mechanism

**Figure 1:**
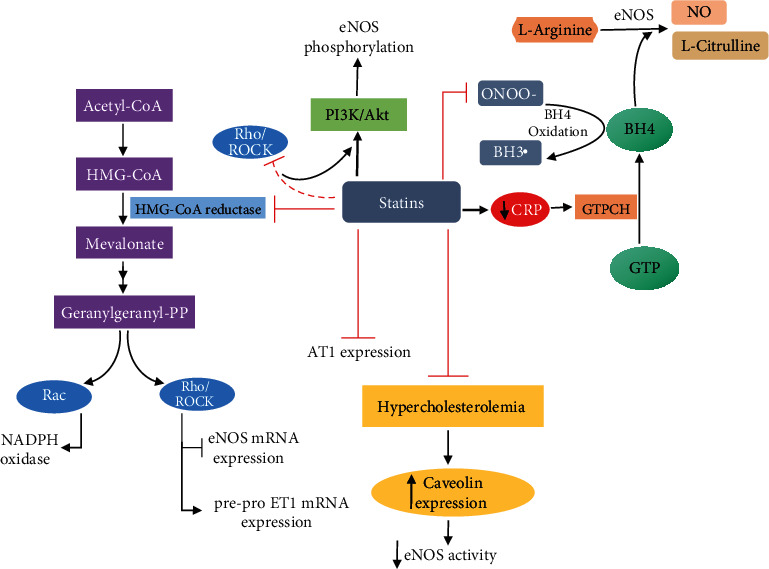
Suggested statin mechanisms of action to improve arterial elasticity. mRNA, RNA messenger; NO, nitric oxide; eNOS, endothelial NO synthase; ET-1, endothelin 1; ROCK, Rho-associated protein kinase; AT-1, angiotensin II receptor type 1; PI3k, phosphoinositide 3-kinase; Akt, protein kinase B; CRP, C-reactive protein; BH4, tetrahydrobiopterin; GTP, guanosine triphosphate.

The results of meta-analyses have suggested that the effect of statins on arterial elasticity appears to be due, at least in part, to their pleiotropic effects, which are independent of changes in blood pressure, lipid profile, and even statin type (Figure 1) [101, 102]. As mentioned above, in addition to their lipid-lowering properties, statins also have pleiotropic effects, such as anti-inflammatory, antiproliferative, antioxidant, and antithrombotic properties. Additionally, statins reduce endothelial dysfunction, improve vascular and myocardial remodeling, and stabilize atherosclerotic plaque [102–104].

The principal mechanism underlying the pleiotropic effects of statins appears to be inhibition of mevalonate synthesis, and subsequently, inhibition of Rho isoprenylation and its downstream target, Rho kinase (ROCK) [102, 103]. Binding of isoprenylated Rho to ROCK leads to ROCK activation, which, in turn, results in a reduction in the half-life of eNOS mRNA and NO production. Thus, statins can induce production of NO by inhibition of the Rho/ROCK pathway [5, 105–108]. Additionally, although it is not clearly understood at this time, it has been demonstrated that statins stimulate activity in the PI3K/AKT pathway, which ultimately leads to eNOS phosphorylation and NO production [109, 110]. Importantly, and as mentioned above, statins can indirectly inhibit the Rho/ROCK pathway, which accelerates the activity of the PI3K/AKT pathway and NO production [111].

In addition, administration of statins is associated with the regulation of paracrine mediators, including angiotensin II and endothelin [3]. In vitro studies have indicated that synthesis of endothelin-1 (ET-1), a potent vasoconstrictor and mitogenic peptide that enhances the tone of vascular smooth muscle cells (VSMCs), and the mRNA expression of its precursor, pre-proET-1, decrease following statin treatment. The reduction in the expression of pre-proET-1 appears to be related to the capacity of the statin to suppress Rho GTPases [112–114]. Angiotensin II (AII) is a vasoconstrictor hormone that contributes to vascular stiffening via an increase in collagen formation, vascular hypertrophy, matrix remodeling, oxidant stress, and decrease in elastin synthesis and NO production [3]. Several studies have revealed that statin therapy downregulates the expression of angiotensin II receptor type 1 (AT1) and mitigates the biological function of Ang II [115–118]. In fact, there is evidence that statins reduce NAD(P)H oxidase activity, a multicomponent enzyme complex that is responsible for ROS generation, by suppressing the geranylation of Rac GTPase and downregulating other NAD(P)H oxidase subunits [118–122].

Another mechanism of statin action is related to the regulation of tetrahydrobiopterin (BH4) concentration, which is an essential cofactor of eNOS. Statins can increase the availability of BH4 either by preventing the oxidation of BH4 to BH3 radical or by reducing C-reactive protein (CRP), an inflammatory marker that can block the activity of the rate-limiting enzyme GTP cyclohydrolase-1, which is needed for the synthesis of BH4 [123]. Additionally, it has been demonstrated that hypercholesterolemia reduces the activation of eNOS by increasing caveolin expression and that statin therapy leads to a suppression in the increased expression of caveolin, which subsequently improves NO synthesis [124–126].

Lastly, previous studies have shown that statins can reduce arterial stiffness by several other mechanisms, which include (1) inhibition in the migration of monocytes [127], (2) modification of the inflammatory response of macrophages and endothelial cells [128], (3) downregulation in ICAM-1 expression [118], (4) reduction in elastin degradation (i.e., greater elastin in statin treated animals) [129], (5) reduction in collagen formation (i.e., since collagen imparts stiffness to the myocardial wall) [119, 130, 131], (6) inhibition of atherosclerosis progression, and (7) inhibition of VSMC proliferation [132].

## 10. Conclusion

In recent years, arterial stiffness as an early marker of cardiovascular disease and mortality has attracted the attention of a large number of investigators. It has been reported that each 47 cm/s increase in pulse-wave velocity is associated with a 5-year increase in arterial age [133], and 1 m/s increase is associated with a 13% increase in lethal cardiovascular events death [134]. Therefore, finding an effective approach to improve vascular elasticity is a priority. Several studies have been performed to assess the efficacy of HMG-CoA reductase inhibitors in improving arterial stiffness. Although several studies showed positive effects of statins on vascular elasticity, some others reported conflicting results. This discrepancies may be due to small sample size, heterogeneous populations, short durations of follow-up, absence of a placebo group, inadequate dose of statins, and the lack of controlling for potential confounding factors. Considering the statins' pleiotropic effects, to provide mechanistic perspective about the role of statins on vascular elasticity, concomitant assessment of antioxidant markers and inflammatory parameters such as high-sensitivity C-reactive protein could have been helpful for future studies.

## Figures and Tables

**Table 1:** List of clinical trials reporting the effects of statin therapy on arterial stiffness parameters.

Author, year	Study design	Interventions	Statin dose	Duration	Population	Number of participants	Method of arterial stiffness assessment	Main finding
Wallace et al., 2010 [35]	Randomized, double-blind, placebo-controlled	1. Simvastatin 2. Placebo	40 mg/d	14 days	Healthy volunteers aged 20–40 years	50	Aortic and brachial PWV, AIx, and AP	Compared with placebo, pretreatment with simvastatin significantly suppressed the enhancement of aortic PWV following typhoid vaccination.
John et al., 2015 [36]	Randomized, double-blind, placebo-controlled	1. Simvastatin 2. Placebo	20 mg/d	6 weeks	COPD patients	64	Aortic PWV	Compared to placebo, arterial stiffness significantly reduced in patients with a high baseline aortic PWV.
Kurpesa et al., 2004 [37]	Randomized, placebo-controlled	1. Simvastatin 2. Placebo	20 mg/d	6 months	Individuals with primary arterial hypertension and high-normal serum cholesterol level	52	PWV	PWV lowered in simvastatin group compared to baseline
Ballard et al., 2015 [38]	Randomized, double-blind, cross-over	1. Simvastatin 2. Placebo	20 mg/d	8 weeks	Patients with a history of statin myalgia	100	Central PWV, peripheral PWV, AIx, and AP	Central PWV improved in simvastatin treatment group.
Shige et al., 2001 [39]	Randomized, double-blind cross-over	1. Simvastatin 2. Placebo	20 or 40 mg/d	4 weeks	Hypercholesterolaemic patients	20	Central and peripheral PWV, arterial compliance	Peripheral PWV significantly improved following simvastatin treatment.
Mäki-Petäjä et al., 2007 [40]	Randomized double-blind cross-over	1. Simvastatin 2. Ezetimibe	20 mg/d	6 weeks	Patients with active rheumatoid arthritis	20	Aortic PWV, AIx, and AP	There was substantial reduction in PWV following both treatments compared to baseline.
Efrati et al., 2007 [41]	Nonrandomized	1. Simvastatin 2. Ezetimibe 3. Ezetimibe + simvastatin (40 mg/d)	40 mg/d 80 mg/d	3 months	Hypercholesterolemic patients	40	AIx	A marked reduction was observed in the simvastatin 40 mg group.
Gepner et al., 2019 [42]	Randomized, double-blind, placebo-controlled	1. Simvastatin 2. Placebo	40 mg/d	12 months 18 months	Middle-aged adults at increased risk for dementia	88	AIx	A transient improvement was observed at 12 months.
Balaguer et al., 2016 [43]	Randomized, double-blind, placebo-controlled	1. Simvastatin 2. Placebo	40 mg/d	12 weeks	Stable COPD	18	PWV, AIx	No significant changes were observed.
Cash et al., 2013 [44]	Randomized, single-blind, placebo-controlled	1. Simvastatin 2. Placebo	20 mg/d	12 months	Primary biliary cirrhosis patients with hypercholesterolaemia	13	Aortic PWV, AIx75, and Tr	No significant changes were observed.
Wang et al., 2012 [45]	Single-arm	Atorvastatin	20 mg/d	6 months	Hypertensive patients	73	baPWV	baPWV was reduced significantly compared with baseline.
Shinohara et al., 2005 [46]	Single-arm	Atorvastatin	10 mg/d	6 months	Type-2 diabetic patients with hypercholesterolemia	22	Femoral-ankle PWV, heart-carotid PWV, heart-brachial PWV, and heart-femoral PWV	A reduction in femoral-ankle PWV was perceived.
Castejon et al., 2017 [47]	Single-arm	Atorvastatin	20 mg/d	8 weeks	Systemic lupus erythematosus females	37	Aortic PWV	PWV significantly improved in patients with baseline pathological arterial stiffness.
Van Doornum et al., 2004 [48]	Single-arm	Atorvastatin	20 mg/d	6 weeks 12 weeks	Patients with rheumatoid arthritis	29	AIx	AIx was decreased by the treatment.
Ratchford et al., 2011 [49]	Single-arm	Atorvastatin	80 mg/d	14 days 30 days	Stroke-free and statin-naive subjects over age 45	40	SI	SI significantly reduced at day 30.
Kontopoulos et al., 2002 [50]	Single-arm	Atorvastatin	20 mg/d	2 years	Hypercholesterolemic patients	36	SI	Aortic SI was significantly reduced by 14%.
Leibovitz et al., 2001 [51]	Single-arm	Atorvastatin	Starting dose: 10 mg/d (mean dose: 16 ± 1.6 mg/d)	20 weeks	Patients with severe hypercholesterolemia	17	Arterial compliance	Small artery compliance was significantly increased with treatment.
Leibovitz et al., 2003 [52]	Single-arm	Atorvastatin	10-20 mg/d	3 months	Hypertensive hyperlipidemic patients	21	Arterial compliance (small and large arteries)	Small artery compliance significantly elevated by atorvastatin treatment.
Akgullu et al., 2008 [53]	Single-arm	Atorvastatin	40 mg/d	6 months	Patients with coronary artery disease	33	Small and large arteries compliance	Arterial compliance significantly improved after treatment.
Yamaguchi et al., 2010	Single-arm	Atorvastatin	20 mg/d	6 months	Type-2 diabetic patients with high non-HDL-C levels receiving atorvastatin (10 mg/day)	39	CAVI	CAVI declined substantially by 4.6%.
Tulmaç et al., 2012 [54]	Single-arm	Atorvastatin	80 mg	Acute	Dyslipidemic patients without atherosclerosis	30	RI, SI	Arterial stiffness did not improve with the treatment.
Ozaki et al., 2006 [55]	Single-arm	Atorvastatin	10 mg/d	6 months 12 months	Hypercholesterolemic patients	30	baPWV	PWV was unaffected by the treatment.
Grigoropoulou et al., 2019 [56]	Nonrandomized	1. Atorvastatin (+Mediterranean diet) 2. Mediterranean diet	10 mg/d	3 months 6 months 12 months	Subjects with type-2 diabetes and dyslipidemia	79	Aortic PWV, carotid-radial PWV	Aortic PWV significantly improved in simvastatin group compared to diet alone.
Meng et al., 2009 [57]	Nonrandomized	1. Atorvastatin (+low-fat diet) 2. Low-fat diet	10 mg/d	3 months 6 months	CAD patients with hyperlipidemia	63	Aortic PWV, carotid-radial PWV, and carotid-distal PWV	Compared with the control group and baseline, PWV substantially decreased after 6 months.
Huang et al., 2014 [58]	Randomized	1. Atorvastatin (in combination with levamlodipine besylate) 2. Levamlodipine besylate treatment	20 mg/d	8 weeks	Hypertensive patients with dyslipidemia and body's inflammatory response	120	PWV	Atorvastatin significantly improved PWV compared to the levamlodipine besylate-treated group.
Orr et al., 2009 [59]	Randomized, double-blind, placebo-controlled	1. Atorvastatin 2. Placebo	80 mg/d	12 weeks	Overweight and obese middle-aged and older adults	26	Aortic and carotid-radial PWV, SI, and arterial compliance	Aortic PWV, SI, and arterial compliance improved compared to placebo.
Kanaki et al., 2013 [60]	Randomized, double-blind, placebo-controlled	1. Atorvastatin 2. Placebo	10 mg/d	6 months	Hypertensive and hypercholesterolemic patients	50	Aortic PWV, normalized PWV, AIx75, and AP	Arterial stiffness parameters significantly improved compare with placebo.
Manisty et al., 2009 [61]	Randomized, single-blind	1. Atorvastatin 2. Placebo	10 mg/d	12–18 months	Hypertensive patients	142	Carotid AIx, AP, and RI	Carotid AIx, AP, and RI from the body significantly improved compared to placebo.
Davenport et al., 2015 [62]	Randomized, open-label	Atorvastatin	10 mg/d 80 mg/d	3 months 12 months	Male patients with type-2 diabetes	51	Aortic PWV	PWV decreased at 3 months and 12 months.
Lee and Kim, 2008 [63]	Randomized	Atorvastatin	10 mg/d 40 mg/d	One week 8 weeks	Patients with moderate cholesterolemia	51	Carotid-radial PWV	PWV improved after 8 weeks of 40 mg atorvastatin treatment, and a significant difference was observed between the groups.
Tousoulis et al., 2013 [64]	Randomized, double-blind, cross-over	Atorvastatin	10 mg/d 40 mg/d	4 weeks	Ischemic heart failure patients	22	AIx75	Compared with the low-dose group, 40 mg/d atorvastatin treatment significantly improved AIx75.
Karter et al., 2003 [65]	Randomized, double-blind	Atorvastatin	20 mg/d 80 mg/d	3 months	Hypercholesterolemic patients	20	Distensibility coefficient	There was noticeable increased in distensibility coefficient of both groups.
Mukherjee et al., 2008 [66]	Randomized, placebo-controlled	1. Atorvastatin 2. Placebo	10 mg/d	6 months	Normotensive normolipidemic persons with type-2 diabetes	57	baPWV	baPWV improved significantly in the atorvastatin group compared with baseline.
Kabaklić and Fras, 2017 [67]	Randomized, double-blind, placebo-controlled	1. Atorvastatin 2. Placebo	20 mg/d	3 months 6 months	Patients with angina pectoris and normal coronary angiogram	58	AIx, AIx75	No marked change was perceived.
Fassett et al., 2010 [68]	Randomized, double-blind, placebo-controlled	1. Atorvastatin 2. Placebo	10 mg/d	3 years	Patients with CKD	37	Aortic PWV, AIx, arterial pressure	No significant changes were observed.
Joyeux-Faure et al., 2014 [69]	Randomized, double-blind, placebo-controlled	1. Atorvastatin 2. Placebo	40 mg/d	12 weeks	Obstructive sleep apnea syndrome patients	51	Aortic PWV	PWV did not alter compared to the placebo group.
Hong et al., 2013 [70]	Randomized, double-blind, cross-over, placebo-controlled	1. Atorvastatin 2. Placebo	40 mg/d	6 weeks	First-degree relatives of patients with premature coronary artery disease patients with endothelial dysfunction	35	AIx	No marked difference was observed.
Raison et al., 2001 [71]	Randomized, double-blind, placebo-controlled	1. Atorvastatin 2. Placebo	10 mg/d	12 weeks	Patients with hypertension and hypercholesterolemia	23	Aortic PWV, carotid AIx	No significant difference was perceived between the groups.
Cohn et al., 2009 [72]	Randomized, double-blind, placebo-controlled	1. Atorvastatin 2. Placebo	10 mg/d	8 weeks	Patients with concomitant hypertension and dyslipidemia	325	Small artery compliance, large artery compliance	No significant changes were observed.
Mikhin et al., 2016 [73]	Single-arm	Rosuvastatin	10-40 mg/d	1.5 years	Arterial hypertensive patients with high and very high cardiovascular risk	114	CAVI, AIx	CAVI and AIx were improved by rosuvastatin treatment, 12% and 17%, respectively.
Canepa et al., 2018 [74]	Single-arm	Rosuvastatin	5-10 mg/d	3 months	Newly diagnosed heterozygous familial hypercholesterolemia	20	Aortic PWV, AIx, AIx75, and arterial BP	PWV significantly decreased.
Ikdahl et al., 2015 [75]	Single-arm	Rosuvastatin	20-40 mg/d	18 months	Patients with inflammatory joint diseases	85	Aortic PWV, AIx	None of the arterial stiffness indices substantially altered.
Ikdahl et al., 2016 [76]	Single-arm	Rosuvastatin	20-40 mg/d	18 months	Patients with inflammatory joint diseases	89	Aortic PWV, AIx	AIx and aPWV were noticeably decreased.
Timár et al., 2013 [77]	Single-arm	Rosuvastatin	20 mg/d	6 months	Patients with systemic sclerosis	28	Aortofemoral PWV, carotid-femoral PWV	Arterial stiffness was not affected by the treatment.
Deguchi et al., 2014 [78]	Single-arm	Rosuvastatin	5 mg/d	12 months	Dyslipidemic patients with cerebral infarction	17	CAVI	CAVI showed no substantial change after rosuvastatin therapy.
Pirro et al., 2007 [79]	Randomized open-label	1. Rosuvastatin (with low-fat diet) 2. Low-fat diet	10 mg/d	4 weeks	Patients with primary hypercholesterolemia	71	Aortic PWV	PWV decreased significantly with rosuvastatin compared to baseline.
Igase et al., 2012 [80]	Randomized, open-label	1. Rosuvastatin (+diet and exercise therapy) 2. Diet and exercise therapy	2.5 mg/d	3 months 12 months	Postmenopausal women with dyslipidemia	51	baPWV, SI	Arterial stiffness markedly reduced compared to placebo after 3 and 12 months.
Ott et al., 2012 [81]	Randomized, double-blind, placebo-controlled, cross-over	1. Rosuvastatin 2. Placebo	10 mg/d	42 days	Hypercholesterolemic patients	29	AIx, AIx75	AIx75 was substantially lower compared to the placebo.
Mitsiou et al., 2018 [82]	Randomized	Rosuvastatin	1. Low dose (5 mg/d) 2. High dose (20-40 mg/d)	6 months	Patients with optimally controlled arterial hypertension	40	Aortic PWV, AIx75	Arterial stiffness significantly improved with both treatments, but the high dose was more effective on PWV improvement.
Wang et al., 2014 [83]	Clinical trial	1. Rosuvastatin 2. Nonstatin treatment	10 mg/d	12 weeks	Hyperlipidemic patient without hypertension	120	Radial artery AIx, baPWV	Rosuvastatin significantly decreased the levels of baPWV and radial AIx compared to baseline.
Tam et al., 2011 [84]	Randomized, double-blind placebo-controlled	1. Rosuvastatin 2. Placebo	10 mg/d (starting with 5 mg/d)	12 months	Patients with rheumatoid arthritis	42	AIx	No noticeable change was observed.
Kim et al., 2018 [85]	Single-arm	Pitavastatin	4 mg/d	3 months	Hypercholesterolemic patients	48	Stiffness index-*β*2, SI	Stiffness index-*β*2 improved substantially.
Miyashita et al., 2009 [86]	Single-arm	Pitavastatin	2 mg/d	12 months	Type-2 diabetics patients with hyperlipidemia	45	CAVI	CAVI was reduced significantly.
Muramatsu et al., 1997 [87]	Single-arm	Pravastatin	10 mg/d	6 months	Hypercholesterolemic patients	59	PWV	PWV showed significant improvement in patients with a higher reduction in TC (≥15%) compared with the group with <15% reduction in TC. Reduction in PWV remained over a 5-year period.
Matsuo et al., 2005 [88]	Quasiexperimental	1. Pravastatin 2. Control (healthy untreated volunteers)	10 mg/d	6 months	Hyperlipidemic patients	35	baPWV	baPWV significantly reduced by the treatment.
Duan et al., 2014 [89]	Quasiexperimental	1. Pravastatin 2. Control (healthy untreated children)	5 or 10 mg/d	6 months	Children with medium to giant coronary aneurysms due to Kawasaki disease	27	SI	No significant change was observed.
Forbat et al., 1998 [90]	Single-arm	Fluvastatin	40 or 80 mg/d	1 year	Hypercholesterolemic patients	60	Aortic compliance	There was a marked rise in aortic compliance.
Ersoy et al., 2014 [91]	Single-arm	Fluvastatin	80 mg/d	6 months	Dyslipidemic renal transplant recipients	14	Large and small vessel compliances	Arterial compliance parameters did not change.
Ichihara et al., 2002 [92]	Randomized, double-blind placebo-controlled	1. Fluvastatin 2. Placebo	20 mg/d	3 months 6 months	Diabetic ESRD patients with normal serum lipid levels	22	PWV	Fluvastatin significantly improved PWV compared with placebo after 6 months.
Lunder et al., 2011 [93]	Randomized, double-blind placebo-controlled	1. Fluvastatin 2. Placebo	10 mg/d	14 days 30 days	Apparently healthy, middle-aged males	50	PWV, SI	PWV and SI significantly improved by the intervention.
Yokoyama et al., 2005 [94]	Randomized, open-label	1. Fluvastatin 2. Diet	40 mg/d	12 months	Hyperlipidemic patients	40	SI, baPWV	PWV significantly increased in the control group, while decreased in fluvastatin group.
Hongo et al., 2008 [95]	Randomized, single-blind	1. Fluvastatin 2. Bezafibrate	20–40 mg/d	3, 6, and 12 months and 2, 3, 4, and 5 years	Patients with CAD and hyperlipidemia	93	baPWV	After 12 months, baPWV decreased substantially in fluvastatin-treated group compared to bezafibrate-treated group and remained significant until the end of the study.
Drapkina et al., 2012 [96]	Randomized	1. Atorvastatin 2. Rosuvastatin	—	5 weeks	Patients with arterial hypertension and dyslipidemia	82	AIx, AIx75, RI, and SI	A marked reduction was observed in SI.
Simsek et al., 2014	Clinical trial	1. Atorvastatin 2. Rosuvastatin	20 mg/d 10 mg/d	12 months	Hyperlipidemic patients	108	PWA parameters	Rosuvastatin showed a greater ameliorative effect on vascular stiffness than the atorvastatin.
Liu et al., 2013 [97]	Randomized controlled	1. Rosuvastatin 2. Simvastatin/ezetimibe	10 mg/d	8 weeks	Patients with coronary artery disease	36	baPWV	baPWV was significantly decreased in the rosuvastatin group compared to the simvastatin/ezetimibe group.
Ilyukhin et al., 2005 [98]	Clinical trial	1. Atorvastatin 2. Simvastatin	10 mg/g 20 mg/d	6 months	Patients with ischemic heart disease	38	PWV	Atorvastatin and simvastatin treatment was associated with -10.05% and -4.66% reduction in PWV.
Hongo et al., 2011 [99]	Randomized, open-labeled	1. Rosuvastatin 2. Fluvastatin	2.5–5 mg/d 20–40 mg/d	3 months 6 months 12 months	High-risk CVD patients with dyslipidemia	75	baPWV	Both treatments significantly improved arterial elasticity after 12 months, and rosuvastatin was more effective.
Ichihara et al., 2005 [100]	Randomized, single-blind	1. Simvastatin 2. Fluvastatin 3. Pravastatin 4. Nonstatin antihyperlipidemic drug	5 mg/d 20 mg/d 10 mg/d	3 months 6 months 9 months 12 months	Hyperlipidemic hypertensive patients	85	baPWV	After 12 months, fluvastatin treatment was associated with a significant reduction in baPWV.

AIX, augmentation index; AIx75, augmentation index normalized to 75 bpm; AP, augmentation pressure; CAVI, cardio-ankle vascular index; PWA, pulse-wave analysis; PWV, pulse-wave velocity; baPWV, brachial-ankle PWV; RI, reflection index; SI, stiffness index; Tr, transit time of the reflected wave; BP, blood pressure; COPD, chronic obstructive pulmonary disease; CAD, coronary artery disease; CKD, chronic kidney disease; ESRD, end-stage renal disease; CVD, cardiovascular disease.

## Data Availability

This is a review article that does not include original data.
